# Do Textbooks Matter for Reading Comprehension? A Study in Flemish Primary Education

**DOI:** 10.3389/fpsyg.2019.02959

**Published:** 2020-01-21

**Authors:** Jonas Dockx, Kim Bellens, Bieke De Fraine

**Affiliations:** ^1^Centre for Educational Effectiveness and Evaluation, KU Leuven, Leuven, Belgium; ^2^Methodology of Educational Sciences Research Group, KU Leuven, Leuven, Belgium

**Keywords:** textbook, reading comprehension, primary education, autoregression model, change score model

## Abstract

This study assessed whether textbooks affect academic performance and engagement in reading comprehension in primary education in Flanders (Belgium). The data of the Progress in International Reading Literacy Study 2016 and a reassessment of this study in 2018 were used to describe students’ learning progress in reading comprehension and evolution in engagement between the fourth and sixth grade. The sample consisted of 3051 students in 98 schools. The averages of students’ learning progress and engagement were compared for five textbooks by using multilevel autoregression model and multilevel change score models. Contrasts between textbooks in average learning progress and engagement were also estimated. To control for differences between student populations that are educated with the different textbooks, we controlled for student’s socioeconomic status, language and initial academic performance in fourth grade at the student- and school-level. The main hypotheses were that textbooks affect learning progress and reading engagement. This was based on the literature and prior (mainly) cross-sectional research which describe textbooks as playing an important role in the curriculum that is taught to students on a daily basis. The results of both models showed that textbooks do not affect student’s average learning progress in reading comprehension and evolution in engagement between the fourth grade and sixth grade in Flanders. Hence, the hypotheses were rejected.

## Introduction

Textbooks are often thought to guide the teacher’s daily practice and to provide the main teaching material that students experience (Schmidt et al., 1997; Mullis et al., 2012a; Harwood, 2017). Accordingly, textbooks are expected to affect students’ learning progress (Törnroos, 2005). This is especially true in countries that are characterized by educational freedom, where publishers are free to translate the government-set objectives for education into teaching materials that teachers can use (Mullis et al., 2012a). For reading comprehension in primary schools, textbooks also affect the reading material students are educated with during primary education. Hence, it is plausible to expect that a school’s choice of a textbook affects that school’s learning progress in reading comprehension.

Flanders (Dutch-speaking northern Belgium) had the largest decrease in average student ability in reading comprehension between 2006 and 2016 out of all participating countries in the Progress in International Reading Literacy Study in 2016 (PIRLS 2016, Mullis et al., 2017b). This result shocked Flemish policymakers, for reading was traditionally considered a strong aspect of Flemish primary education. The results also showed that Flemish students had relatively low engagement for reading lessons. Furthermore, it could not be inferred from the data of PIRLS 2016 what caused this decrease in literacy achievement. Flanders is characterized by large educational freedom for school and teachers, the government only sets minimum attainment targets. Accordingly, a large responsibility in implementing the curriculum rests with schools and teachers, who often base their daily practices on textbooks. Therefore, a specific hypothesis on the decline of performance in reading comprehension and low engagement was that new textbooks had declined in quality and offered less challenging material for students.

In an effort to grasp possible explanations for the decline in reading literacy achievement, the Flemish government decided to fund a reassessment of the students who participated in PIRLS 2016. The students were reassessed in May 2018 in Grade 6. Accordingly, this allowed us to assess what contributes to learning progress and engagement between the fourth and sixth grade in Flemish primary education. Hence, in this study we tested the hypotheses if textbooks affect learning progress in reading comprehension and evolution in engagement from the fourth grade until the sixth grade. In the following sections, we describe literature on textbooks’ role in implementing a curriculum, prior studies on the effects of textbooks, and what is known about teaching reading comprehension.

## Textbooks as a Step in the Implemented Curriculum

The role textbooks have in how students are educated is typically explained by first distinguishing between the intended curriculum, the implemented curriculum and the attained curriculum (Valverde et al., 2002). The *intended curriculum* is what students are intended to learn by the government. Official documents such as legal documents, government brochures and policy statements describe this intended curriculum. This curriculum is thought to reflect a societal vision of education and the political objectives for education (Robitaille et al., 1993; Schmidt et al., 1997). The *implemented curriculum* is what teachers teach on a daily basis to their students in their classroom. It encompasses teaching practices, classroom management and the teachers’ choices and timing (Robitaille et al., 1993). It is not necessarily equal to the intended curriculum but is a specific interpretation. It affects what learning content is available to students and how the content is taught. Accordingly, students learn from the implemented curriculum, but what competences they achieve, behaviors they perform and attitudes they acquire may differ from what teachers meant. This is the attained curriculum (Eggen et al., 1987). In sum, the intended curriculum, implemented curriculum and attained curriculum correspond, respectively with the official objectives of education, the teacher’s daily practices in education and the student’s results in education.

A textbook and its associated materials has the role of a mediator between the different curricula, it mediates between the intended and implemented curriculum. The textbooks translate the official objectives into a guide for teachers with materials that can be used on a daily basis. However, teachers can still make their own decisions in how to use the textbooks. Teachers can select parts of the textbook and they can decide on how much time is spent on certain topics in the textbook. Hence, a textbook is only a “potentially implemented curriculum” (Schmidt et al., 1997), teachers can still adapt the materials and choose what to offer to their students. Because textbooks are also the main educational material students are subjected to in class, textbooks also mediate between the implemented curriculum and attained curriculum. In sum, textbooks mediate between the intended, implemented and attained curriculum.

Another way to understand the role textbooks have in education is that they affect the opportunity to learn students have (Törnroos, 2005). The opportunity to learn (OTL) is usually defined as the total effective learning time spent on a subject. This consists of the time wherein the teacher explicitly teaches the subject and the time wherein the students study the subject’s learning contents. Furthermore, it includes which learning materials are available for students. Considering that textbooks provide the learning materials and didactic cues for teachers, they are likely to affect the OTL. The textbook also provides teachers with a mental framework on what and how to teach (Nicol and Crespo, 2006). However, exactly how teachers use the textbooks in their daily teaching will differ (Harwood, 2017), so textbooks will not determine the OTL entirely. Rather, textbooks are simply one resource teachers and students can use during the process of education.

The role the textbooks have in the intended curriculum or the OTL is also linked to the concept of educational freedom. In many education systems, there is a free choice in textbooks (Exhibit 7 in Mullis et al., 2012a; van Zanten and van den Heuvel-Panhuizen, 2014) as a result of the educational freedom of schools. This is also the case for Flemish education. In these countries, the power the government has in determining the curriculum the students receive is relatively small. Therefore, it is expected that in such countries, textbooks have relatively larger effects on the intended curriculum or OTL (Törnroos, 2005; Mullis et al., 2012a). This may be especially true for education systems without quality control on the textbooks. In sum, it is plausible to expect that in countries with high educational freedom, differences in academic performance are more likely to be attributable to the different textbooks that are used in different schools.

## Effects of Textbooks

The concepts implemented curriculum and OTL provide theoretical frameworks on how textbooks are hypothesized to influence what teachers do on a daily basis and what students learn. Accordingly, in this section we describe what research shows on the effects of textbooks on teachers’ practice and on students’ academic performance.

In most countries, teachers use textbooks as a basis for their daily teaching for reading comprehension. In PIRLS 2006 it was reported that 74% of primary school teachers across participating countries used a textbook as a basis for teaching. However, differences between countries exist, and the average per country ranged from 20 to 80% (Mullis et al., 2007). In PIRLS 2011 it was reported that 72% of primary school teachers across participating countries used a textbook as a basis for teaching. Again, differences between countries exist, and the average per country ranged from 14 to 98% (Mullis et al., 2012b). Comparable results were found for the usage of textbooks for mathematics in TIMSS (Mullis et al., 2012a). Horsley and Sikorová (2014) also note that from 2003 to 2011, the percentage of teachers who used textbooks as a basis for instruction rose.

Studies have been conducted that assess how a teacher’s behavior is affected by the choice for a textbook. Schmidt et al. (2001) found that the space a topic receives in a textbook positively influences the time spent on that topic during class. Furthermore, whether a topic is included in the textbook affects the probability it will be included during class and the importance teachers ascribe to that topic (Schmidt et al., 1997; Stein et al., 2007). In Sweden, Johansson (2006) also found that textbooks guide which content is presented to students. Lepik et al. (2015) analyzed teacher questionnaires in Estonia, Finland and Sweden. They found that between 49 and 64% of the teachers prepare their lessons based on a textbook, while between 79 and 92% of the teachers mainly use the textbooks as a source for exercises. Note that the effect of a textbook on student’s curriculum is likely only partially mediated by the teacher’s behavior. Rather, textbooks are typically also (part of) the material that is available to students on a daily basis (Schmidt et al., 2001; Mullis et al., 2012a, b). Given that students tend to be unaware of the intended curriculum (Schmidt et al., 1997), the textbook may be perceived by students to be the “official” curriculum. Accordingly, Lepik (2015) found that in Estonia most problems for students’ in-class exercises and homework were taken from mathematics textbooks. In summary it can be stated, that mathematics textbooks are an important and extensively used resource for teaching and students’ learning materials.

Another stream of studies investigated whether the textbook affected students’ academic performance. Törnroos (2005) found in the Finnish TIMSS 1999 data that the textbook used affected students’ performance in mathematics. Specifically, the number of learning opportunities in a textbook was positively correlated with students’ achievement. Accordingly, Hadar (2017) assessed whether textbooks that provide opportunities for higher levels of understanding in mathematics affect students’ performance in Israel’s Arab community. They found that these opportunities positively affected students’ performance. In a German longitudinal study from grade 1 to grade 3, van den Ham and Heinze (2018) showed that the mathematics teachers’ textbook choice had a substantial effect on the students’ mathematics achievements. This effect remained even when the authors controlled for both student background variables and teachers’ background variables at the start of the study.

In Flanders, Van Steenbrugge et al. (2013) assessed how textbooks affect mathematics teachers and students’ academic performance in mathematics. In the first part of their study, the authors found that there are differences between textbooks in how well they were liked by the 841 teachers in their sample. In the second part of their study, a cross-sectional study of 1579 students in the first grade to sixth grade, no relation was found between textbooks and students’ academic performance. In contrast to the former authors, Goffin et al. (2016) analyzed the effects of textbooks on academic performance in mathematics using cross-sectional national assessment data of 6940 sixth-grade students. The authors found clear differences between textbooks, even after controlling for socioeconomic background indicators at the school-level. Accordingly, a cross-sectional study in grade 4 on the Flemish TIMSS 2015 data (Bellens et al., 2019) showed that textbooks are related to the average academic performance in mathematics. Furthermore, even after controlling for student characteristics, differences between textbooks remained. Notably, both Goffin et al. (2016) and Bellens et al. (2019) found similar patterns for which specific textbooks were more effective for mathematics than others.

In sum, most evidence indicates that textbooks relate to teachers’ daily teaching, both in teaching content and how it is taught, and that textbooks are related to student’s academic performance. Accordingly, this supports the view that textbooks have a key role in the implemented curriculum and attained curriculum. However, most studies on the effects of textbooks used cross-sectional data, whereas having longitudinal data is a necessary condition to describe learning gains of students and schools, and make causal conclusions on the role of textbooks (Singer and Willett, 2003). Moreover, studies on the effects of textbooks have mainly focused on performance in mathematics. There have been no prior studies on academic performance in reading comprehension or reading engagement.

## Textbooks and Reading Comprehension

There are substantial reasons to expect that textbooks would also influence students’ performance in reading comprehension and engagement. In this section, we describe how the ability of reading comprehension is defined, how teaching practices can help students’ development in reading comprehension and engagement and how these can be related to textbooks.

Recently, most authors agree that reading comprehension is a reader’s active construction of meaning based on the interaction between the reader and the text, within the context of a particular reading experience (Snow and RAND Reading Study Group, 2002; National Center for Educational Statistics, 2005; Mullis and Martin, 2015). In PIRLS (Mullis and Martin, 2015), the construction of meaning is considered to be the result of a complex interplay between several abilities, which include linguistic skills, cognitive strategies, metacognitive strategies and background knowledge. Defining reading comprehension as the active construction of meaning is derived from several theoretical frameworks (Graesser, 2007). Broadly, we can distinguish between frameworks that focus on how knowledge from different text elements are integrated into one mental representation (Kintsch, 1998), and frameworks that focus on how readers use reading strategies to construct meaning (Graesser et al., 1994; Zwaan, 1999). These different frameworks agree that the active construction of meaning is key to reading comprehension, but they differ on the role of strategies and how the construction of meaning happens.

The definition of reading comprehension as a reader’s active construction of meaning differs from the “simple view of reading comprehension” (Hoffman, 2011). In this view reading comprehension is the sum of decoding skills (letter-sound correspondence rules) and listening comprehension (e.g., Gough and Tunmer, 1986; Hoover and Gough, 1990). This theory suggests that decoding and vocabulary are initially the most important, but that language comprehension becomes more important afterward (e.g., Cutting and Scarborough, 2006; Aaron et al., 2008; Keenan et al., 2008). This view has some merit, inaccurate word decoding can hinder reading comprehension (Lyon, 2002), but it is incomplete. The simple view cannot explain how readers can construct meaning from textual propositions, can employ different reading strategies for different texts, how experience in reading may benefit other language skills, and how readers deal with the specific structures of texts that are not encountered elsewhere (Oakhill et al., 2003; Kendeou et al., 2009). Hence, the simple view is incomplete.

Like the definition of reading comprehension, how reading comprehension should be taught has been approached in different ways. These approaches seem related to children’s ages, so each approach contributes a different viewpoint how reading comprehension should be taught. Hence, in the following paragraphs we describe these different approaches to teaching reading comprehension.

At a young age, children should develop basic decoding skills, word-specific knowledge (e.g., Lonigan et al., 2000; Roth et al., 2002; Storch and Whitehurst, 2002; Kirby et al., 2003; Muter et al., 2004; Lesaux et al., 2007) and listening comprehension. At a young age, children’s reading comprehension is still strongly related to listening comprehension and technical reading ability (Panel, 2000; Pressley et al., 2001; Kintsch, 2004; Van Gelderen et al., 2004, 2007; Korpipää et al., 2017). Teachers should also give attention to world knowledge, story structure and vocabulary (Panel, 2000; Duke et al., 2011). However, teachers should also spend time on reading comprehension itself, for reading comprehension develops simultaneously with the ability in technical reading. The construction of meaning should stay within the realm of the young children’s world and require a limited number of inferences, for young children will still need a lot of resources for technical reading (Fuchs et al., 2001; Glenberg et al., 2004; Pikulski and Chard, 2005; De Koning and van der Schoot, 2013).

When children have become proficient in decoding, attention should shift toward teaching reading strategies and expanding the children’s vocabulary (Pressley et al., 2001; Bimmel and Van Schooten, 2004; Afflerbach et al., 2008). Children should be consistently engaged, but with a gradual shift in responsibility for the success of reading from the teacher to the child (Boardman et al., 2018). This process starts with a teacher demonstrating and explaining his thought processes during reading to serve as a model for children (Schunk, 2003; Afflerbach et al., 2008; Hock et al., 2015). Afterward, the individual student gradually attains more autonomy, a process called “scaffolding” (Pressley et al., 2001; Alvermann, 2002; Nokes and Dole, 2004; Fisher et al., 2008; Rupley et al., 2009). During this process children are consistently made aware of which strategy fits best in which situation and how to steer these strategies (Baker and Scher, 2002; Pearson and Cervetti, 2013, p. 531; Wilkinson and Son, 2011). The shift from the teacher to child also allows the usage of teaching methods where the children have more responsibility (Nokes and Dole, 2004; Duke et al., 2011;, p. 167). Texts should slowly change from a real-life situation in the children’s world to a more abstract representation (Glenberg et al., 2004; Alexander and Fox, 2011; De Koning and van der Schoot, 2013; Guan et al., 2013).

At a later stage, children should also be supported to develop their engagement and motivation for reading so they can autonomously develop their reading skills (De Naeghel et al., 2016). This is especially important at the end of primary education and during secondary education when students’ engagement and motivation for reading generally declines. Note that from secondary education onward, language will often be specific to a certain discipline (Shanahan, 2017). Therefore, a focus on only general strategies for reading comprehension may no longer be sufficient in areas with a specific nomenclature, syntax and text structure (Hirsch, 2006; Moje, 2008; Smagorinsky, 2015).

In sum, ability in reading comprehension can be influenced by how decoding skills, general language comprehension and reading strategies are taught. Furthermore, when children get older, it becomes important to keep students engaged and motivated to focus on reading. Given that textbooks are expected to affect which content is taught and give didactic cues to teachers, it can be expected that textbooks influence students’ development in reading comprehension and engagement.

## Current Study

The goal of this study was to investigate if the use of different textbooks for reading (comprehension) in primary education matters for students’ learning progress in reading comprehension and engagement. This research question is derived from concerns in Flanders that, because of the combination of educational freedom and a lack of control on textbook quality, some textbooks may hinder learning progress in reading comprehension and engagement in reading. Furthermore, in prior studies the effects of textbooks have been mainly investigated for mathematics but not yet for reading comprehension and engagement.

Primary education in Flanders is compulsory and is meant for students from age 6 to 12. Consequently, most students spend 6 years in primary education in age-groups until they are 12 years old. Before primary education the vast majority of students will have spent three to 4 years in pre-primary education. Attainment targets are set by the government, these determine the minimum goals that all students should achieve at the end of primary education. The governing boards of schools are responsible for the development of their own curriculum. In practice, most schools are part of a school association (e.g., the association of catholic schools, the association of government-mandated schools, and the association of school of the municipalities) and this school association develops a curriculum. Therefore, it is correct to state that there are multiple “national curricula” present in Flanders. However, each school adapts the curriculum to their pedagogical vision and student population. There is a large emphasis on educational freedom in Flanders, and schools are free to determine how to teach. Furthermore, because the attainment targets are only minimum goals, schools are free to embellish the learning content in ways they see fit. Similarly, publishers have a large freedom in developing textbooks, there is no governmental control on the development of textbooks. Schools can freely choose between the available textbooks.

The research question of this study was if the usage of different textbooks for reading (comprehension) in primary education matters for students’ learning progress in reading comprehension and evolution in engagement for reading. Accordingly, our hypothesis was that textbooks make a difference in learning progress and engagement. To test this hypothesis, we used data from PIRLS 2016 and the reassessment of the same students 2 years later. Achievement in reading comprehension and engagement for reading were assessed at both time points, this allows us to describe the progress during the last 2 years of Flemish primary education.

We estimated the effects of different textbooks by a multilevel model, with students nested in schools. Textbooks are a school-level variable and are used as an explanatory dummy-coded variable. Because the textbooks that schools use are related to the background characteristics of their students, any estimated effect of textbooks would be biased if we did not account for the differences in student background characteristics. Therefore, models were also used which incorporated students background characteristics as controls. In the following section, the sample and methods are described in more detail.

## Materials and Methods

### Sample

We used the Flemish data from PIRLS 2016 and data from the schools that participated in a reassessment of PIRLS in 2018. In 2016, data were collected from 5,198 students in 148 schools. Alongside the achievement tests that measured students’ ability in reading comprehension, student questionnaires, teacher questionnaires, principal questionnaires and parent questionnaires were administered to gather information on the students’ learning environments. Engagement for reading was part of the student’s questionnaire. Instruments were in Dutch, the official language in Flanders.

During the reassessment of PIRLS in 2018, students in 126 of the 148 schools of the original sample from PIRLS 2016 were reassessed. Note that the Flemish PIRLS 2016 sample had 7 schools for special education. These schools were not asked to participate in the reassessment in 2018. Hence, our inferences are limited to non-special schools. 15 of the 141 non-special schools could not participate in the reassessment for various reasons. The students who were assessed in 2018 were in the sixth grade, the last year of primary education in Flanders. 4,869 students of the 126 schools participated in the reassessment. 4,046 of these students participated in both PIRLS 2016 and the reassessment in 2018. The reason for this lower number is that a relatively high number of students either change school during the last 2 years of primary education or go to secondary education early. Students who go to secondary education early are mainly low-performing students who go into the remedial track of secondary education. Furthermore, we could only assess the effects of a textbook if a sufficient number of schools used a textbook. Five textbooks were used by a sufficient number of schools. Hence, for 995 students (24.59%) in 28 schools (22.22%), the teacher did not use one of the five most-used textbooks. These could not be included in the analyses, for the sample size was too small per category. Accordingly, these students were removed from the dataset, resulting in the final dataset with 3,051 students in 98 schools. Hence, our inferences are for students who stayed in the same school from the fourth grade until the sixth grade and used one of five most-used textbooks.

Because the 126 primary schools in the reassessment of PIRLS in 2018 participated on a voluntary basis, we assessed whether this sample of schools differed from the population of Flemish primary schools. We used a range of school characteristics: the percentage of students whose mother is lowly educated, the percentage of students whose parents receive an governmental grant due to their low income, the percentage of students who speak another language than Dutch, the province where the school is located, and the educational network of the school (Catholic or official). We found that the sample did not tangibly differ from the population. Accordingly, including sampling weights in our results did not alter our results. Hence, we decided to only present the results of the unweighted analyses.

We note that the data collection of the reassessment of PIRLS in 2018 was carried out by the same research team that collected the PIRLS 2016 data in Flanders. The data collection of the reassessment in 2018 was performed according to the same guidelines as PIRLS 2016.

### Dependent Variables

#### Reading Comprehension

Students’ performance in reading comprehension in 2016 is described by the five plausible values representing the students’ achievement distribution. Students’ performance in reading comprehension in 2018 is also described by five plausible values representing students’ achievement distribution. These five plausible values per students reflect the unreliability in the student’s ability estimate. Because this unreliability is reflected in the five plausible values, it is possible to procure unbiased estimates standard errors, which would not be possible with point estimates. The average of the measurement scale of PIRLS was set at 500 with a standard deviation of 100 across the participating countries in 2001. Note that in 2016 Flanders had an average of 526 with a standard deviation of 60.01. In line with the guidelines by von Davier et al. (2009), all five plausible values were used in the analyses, using techniques for multiple imputations. Analyzing plausible values as if they are multiple imputations yields unbiased and efficient estimates, because the plausible values of a student’s ability are multiple imputations of an unknown true ability (Rubin, 1987; von Davier et al., 2009 p. 11, p. 36; Rutkowski et al., 2013, p. 83, p. 150, p. 165, p. 434; Foy and Yin, 2017, p. 1).

For the reassessment of PIRLS in 2018 in Flanders, the same booklet approach was used as in PIRLS 2016. Whereas in PIRLS 2016 there were 16 booklets, the reassessment in 2018 had 10 booklets. In PIRLS 2016 each booklet consisted of two texts with accompanying questions. The reassessment in 2018 had six booklets that also consisted of two texts with accompanying questions from PIRLS 2016. There were also four booklets which had only one text of PIRLS 2016, but also had two shorter texts. These new texts with accompanying questions were more difficult and their inclusion was to tailor the difficulty level of the booklets to the ability level of the students in Grade 6. Booklets were allocated so that students never received a text in 2018 that they already had read in 2016. An overview of the booklets with their included texts is given in Table 1.

**TABLE 1:** Booklets and texts PIRLS 2018 in Flanders.

**Book**	**Part 1: included text(s)**		**Part 2: included text(s)**	
1	Macy and the Red Hen		Horoscope (new)	Postage stamp (new)
2	Sharks		Airplane (new)	Postage stamp (new)
3	The magic key (new)	Difficult duel (new)	The Green Sea Turtle’s Journey of a Lifetime	
4	Airplane (new)	Postage stamp (new)	Flowers on the Roof	
5	Icelandic horses		Macy and the Red Hen	
6	Oliver and the Griffin		Sharks	
7	The Green Sea Turtle’s Journey of a Lifetime		Oliver and the Griffin	
8	Flowers on the Roof		Icelandic horses	
9	The magic key (new)	Horoscope (new)	Icelandic horses	
10	Difficult duel (new)	Horoscope (new)	Oliver and the Griffin	

#### Engagement

Students’ engagement in reading in PIRLS 2016 is described by the “Students Engaged in Reading Lessons Scale (code in PIRLS 2016: ASBGERL).” Students’ scores on this scale were inferred from the student’s answers on nine statements in a student questionnaire. These statements included “I like what I read about in school” and “My teacher lets me show what I have learned” (for all statements, see Mullis et al., 2017a, Exhibit 10.1, pp. 291–293). The students indicated to what extent they agreed with the statements by ticking “Agree a lot,” “Agree a little,” “Disagree a little” or “Disagree a lot.” Based on a Rasch partial credit model of the students’ answers, the level of each student’s engagement was inferred from that student’s responses to the statements (Martin et al., 2017, Chapter 14, pp. 14.87–14.97). Accordingly, a point-estimate of a student’s underlying engagement was attained by using Warm’s Weighted likelihood estimation (WLE, Warm, 1989). The average of the measurement scale of PIRLS was set at 10 with a standard deviation of 2 across the participating countries in 2016. Note that in 2016 Flanders had an average of 9.5 with a standard deviation of 1.6.

The reassessment of PIRLS in Flanders in 2018 used the same nine statements and the same Rasch partial credit model as in PIRLS 2016 to estimate the level of each student’s engagement. In 2018, the total sample had an average 8.9 with a standard deviation of 1.2.

PIRLS describes engagement as the focus a student has when interacting with the educational content (Mullis and Martin, 2015). Such cognitive engagement is considered a requirement for learning. However, students are subjected to distractions that may make them not engage with the educational content (Yair, 2000). Hence, a teacher’s responsibility is to use methods that maintain students’ engagement during class (Klieme et al., 2009; Lipowsky et al., 2009).

### Independent Variables

#### Textbook

Information on the textbook used by the school was gathered by adding a question in Flanders’ teacher questionnaire. Teachers indicated which textbook they predominantly used, choosing between several options. The five options that are assessed in this study are (English translation between brackets): 1 = “*Kameleon*” (Chameleon), 2 = “*Taalbende*” (Language gang), 3 = “*Taalsignaal*” (Language signal),4 = “*Tijd voor taal*” (Time for language), 5 = “*Totemtaal*” (Totem language). Besides these five textbooks, five other textbooks were also included in the questionnaire. Furthermore, teachers could also indicate if they used another textbook that was not included in the list or they could indicate that they used their own material. However, in this study we only assess the effects of the five most-used textbooks. Textbook was a dummy-coded variable in the analysis. Contrast coding was used, with “*Tijd voor Taal*” as the reference category, for most students are educated with this textbook. Contrasts were estimated for the different textbook dummies.

#### Student Background Variables

Student characteristics were included in our analyses to control for selection bias. Hence, we first investigated which variables available in PIRLS 2016 had high correlations with academic performance in reading comprehension in 2018 and engagement for reading in 2018. Then, we selected those variables that also predicted the usage of specific textbooks. Note that, due to the limited amount of schools (96), it was not possible to include each aggregated student characteristic at the school-level in the model, for this would result in biased estimates (McNeish and Stapleton, 2016b, a; McNeish et al., 2017). Hence, we were looking for the variables with the largest confounding effects (e.g., Greenland and Robins, 2009; VanderWeele and Shpitser, 2013). The remaining variables were assessed for multicollinearity in predicting either academic performance in reading comprehension or engagement in reading. If the VIF was small, indicating low multicollinearity (e.g., O’brien, 2007; Yu et al., 2015), we retained the variable.

Based on this procedure, four student variables were selected: academic performance in reading comprehension in 2016, engagement for reading in 2016, socioeconomic status (SES) and student home language. Below, we describe these control variables. Academic performance in reading comprehension in 2016 and engagement for reading in 2016 were already described in the former section.

Note that, because models are used that partition between school-level variance and student-level variance, student background variables are added at both levels of the models. Accordingly, we are also controlling for the school-level mean of the student background variables when assessing the effects of textbooks.

##### SES

In PIRLS 2016, a “Home resources for Learning” scale was created by means of a partial credit model (Martin et al., 2017). It consists of five items: (a) number of books at home, (b) number of home study supports, (c) number of children’s books in the home, (d) highest level of education of either parent, and (e) highest level of occupation of either parent. The first two were reported by students, the other information was derived from parents’ questionnaires. This scale (with M = 0 and SD = 1) was taken into account in the analyses as an indicator of students’ SES by means of a manifest, continuous variable. In our analyses it was always grand-mean centered.

##### Language

Language spoken at home (LANG) was based on students’ answers in the student questionnaire. Students had to answer a 4-point scale, indicating the extent to which they speak the language of the test (i.e., Dutch) at home, ranging from “never” to “always.” In the analyses, LANG was taken into account as a dummy-variable which indicated either who (0) never or sometimes speak language of a test at home, or (1) always or almost always speak language of test at home (native students). In our analyses it was always grand-mean centered.

### Outcome Analyses: Multilevel Autoregression Model and Multilevel Change Score Model

**FIGURE 1:**
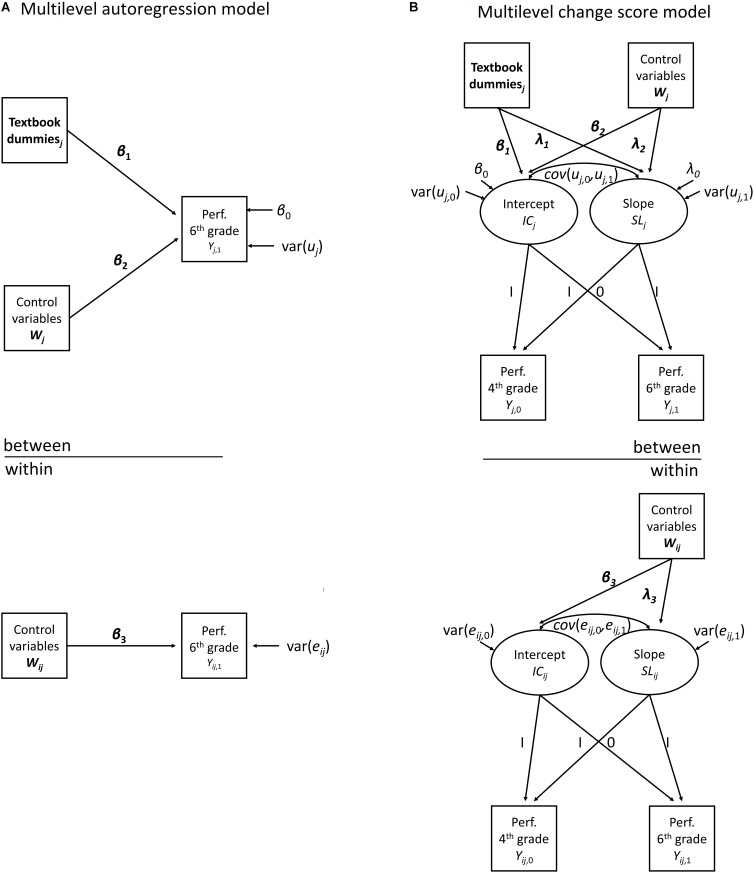
Comparison of multilevel autoregression model and multilevel change score model for assessing the effects of textbooks on learning progress in reading comprehension between fourth and sixth grade.

We used two models to estimate the effect of textbooks on learning progress in reading comprehension and the evolution in engagement from fourth grade to sixth grade. The first, the auto-regression model, is the most-often used. The second, the change score model, is less-often used. Both models were fit as multilevel models to account for the hierarchical structure of the data, with students nested within schools. Therefore, the variance in the outcome is partitioned in two parts: between-school variance and within-school variance. This partitioning of the variance is achieved by the inclusion of shared residual for all students in the same school, which is the heterogeneity in the error terms (e.g., Hox, 2010; Goldstein, 2011; Snijders and Bosker, 2012). Cohen’s *d* was used for effect size interpretation of the estimated contrasts (Cohen, 1977). We describe the multilevel autoregression model and the multilevel change score model in the following paragraphs. The graphical representation of both models is shown in Figure 1.

#### The Multilevel Autoregression Model

In an autoregression model the academic performance in reading comprehension (or students’ engagement) in the sixth grade is regressed on academic performance in reading comprehension (or students’ engagement) in the fourth grade. The main rationale of this model is that by accounting for the prior achievement (or earlier level of engagement), any effect of textbooks on academic performance in reading comprehension (or engagement) in sixth grade cannot be attributed to differences between textbooks in academic performance in reading comprehension (or engagement) in fourth grade (McArdle, 2009; Castro-Schilo and Grimm, 2018).

The multilevel autoregression model was developed in several steps. We started with an empty model, the null model, without explanatory variables, in which the total variance in reading comprehension (or engagement) in sixth grade was partitioned between the school-level and student-level. In the next model, Model 1, we included textbook as an explanatory variable at the school-level for describing raw differences between textbooks in reading comprehension (or engagement) in sixth grade. In Model 2, we added reading comprehension (or engagement) in fourth grade as an explanatory variable at the school-level and student-level to control for prior differences between textbooks in reading comprehension (or engagement) in sixth grade. Next, in Model 3, we added the student background characteristics at the school-level and student-level to control for textbooks being used in schools with different student populations. Model 3 for academic performance in reading comprehension is shown in Eq. 1:

(1) $$Y_{i\,j,1}=\beta_{0}+\mathrm{Textbook}\,{\mathrm{dummies}}_{j}\,\beta_{1}+W_{j}\,\beta_{2}+W_{i\,j}\,\beta_{3}+u_{j}+e_{i\,j}$$

*Y*_*ij*_ is the performance of person *i* in school *j* for ASRREA18. *Y*_*ij*_ is a function of: parameter β_0_, the overall intercept, the mean of the scores on *Y*_*ij*_ across all schools when all the predictors have a zero value; a vector of parameters **β***_1_* and dummy-coded variable ***Textbook dummies_*j*_***, describing the average change in performance compared to the intercept if school *j* uses a textbook different from the reference textbook; ***W****_*j*_***β**_2_, where **β**_2_ is a vector of parameters describing the average change in performance for the standardized values of the covariates in ***W****_*j*_* measured for school *j*.***W****_*ij*_***β***_3_*, where **β**_3_ is a vector of parameters describing the average change in performance for the standardized values of the covariates in ***W*_*ij*_** measured for student *i* in school *j*; *u*_*j*_, the residual for school *j*, and the error term *e*_*ij*_ for person *i* in school *j*. We assume that the level 1 error, *e*_*ij*_, has a univariate normal distribution, *N*(0,$\sigma_{e_{i\,j}}^{2}$), and that the level 2 error, *u*_*j*_, has a univariate normal distribution, *N*(0,$\sigma_{u_{j}}^{2}$).

While the autoregression model (or residualized change score model) has been the standard approach to assess change in average test scores between two time points, recently there has been a renewed interest in the usage of change score models (e.g., Castro-Schilo and Grimm, 2018, pp. 46–48; Eriksson and Häggström, 2014, p. 2; Gollwitzer et al., 2014, pp. 676–678; McArdle, 2009; van Breukelen, 2013, pp. 895–898, 900–903). This renewed interest in change scores comes from limitations of the autoregression model. Using the autoregression model, it has to be assumed that the estimated relation between the pretest performance scores (in our study, reading comprehension and engagement in 2016) and the posttest performance scores (in our study, reading comprehension and engagement in 2018) is an unbiased estimate of the true relation between pretest abilities and posttest abilities. However, this can only be true if measurement error and sampling error do not exist or are negligible. In practice, the estimated correlation coefficient between pretest scores and posttest scores is a biased estimate of the true correlation coefficient between the true pretest abilities and the true posttest abilities. The main result is that by using the autoregression model, differences in pretest scores between, groups are not completely accounted for. This leads to biased estimates of the average learning progress of groups that already differed on the pretest scores.

Note that we also fitted a multivariate multilevel autoregression model, with both reading comprehension and engagement as correlated outcomes. The parameter estimates in this model were not tangibly different from the univariate model parameter estimates. Generally, a multivariate model yields more efficient parameter estimates, but the efficiency gains of using a multivariate model versus a univariate model is a function of the correlation between the outcomes (e.g., Robins and Rotnitzky, 1995; Teixeira-Pinto and Normand, 2009; Grilli et al., 2016). Given that the correlation between reading comprehension and engagement was relatively low, we have no good statistical argument to use a multivariate autoregression model. Hence, we only present the estimates of the univariate autoregression models.

#### The Multilevel Change Score Model

A change score model can be thought of as a latent growth curve model for two time points. Both the academic performance in reading comprehension (or engagement) in the sixth grade and the academic performance in reading comprehension (or engagement) in the fourth grade load equally on an intercept variable. However, only academic performance in reading comprehension (or engagement) in the sixth grade loads on the slope variable. The main rationale of this model is that the differences in the average change score between the textbooks, as described by the slope variable, are the object of interest (McArdle, 2009; Castro-Schilo and Grimm, 2018).

The multilevel change score model was developed in several steps. We started with an empty model, the null model, without explanatory variables, which distinguishes between average initial performance in reading comprehension (or engagement) and the average change score from the fourth until the sixth grade. The variance in both the initial performance (or engagement) and the change score was partitioned between the school-level and student-level. In the next model, Model 1, we included textbook as an explanatory variable at the school-level for describing raw differences in the initial level and change score between textbooks in reading comprehension (or engagement). In Model 2, we added the student background characteristics at the school-level and student-level as predictors of the initial level in academic performance (or engagement) and change score in academic performance (or engagement) to control for textbooks being used in schools with different student populations. Next, in Model 3, the correlation between the initial performance (or engagement) and change score at the student-level and school-level are set to zero. Instead, the initial performance (or engagement) is now also added as a predictor for the change score at the student-level and school-level. The fourth model is shown in Eqs. 2–4:

(2) $$Y_{i\,j,t}=I\,C_{i\,j}+S\,L_{i\,j}\,t$$

$$I\,C_{i\,j}=\beta_{0}+\mathrm{Textbook}\,{\mathrm{dummies}}_{j}\,\beta_{1}$$

(3) $$+W_{j}\,\beta_{2}+W_{i\,j}\,\beta_{3}+u_{j,0}+e_{i\,j,0}$$

$$S\,L_{i\,j}=\lambda_{0}+\mathrm{Textbook}\,{\mathrm{dummies}}_{j}\,\lambda_{1}$$

(4) $$+W_{j}\,\lambda_{2}+W_{i\,j}\,\lambda_{3}+u_{j,1}+e_{i\,j,1}$$

*Y*_*ij,  t*_ is the performance of person *i* in school *j* at time *t*. *IC*_*ij*_ is the performance of person *i* in school *j* at time *0* (fourth grade); *SL*_*ij*_ is the change score (learning progress) of person *i* in school *j* between time 0 and time 1 (fourth grade to sixth grade).

*IC*_*ij*_ is a function of: parameter β_0_, the average intercept across all schools and students; a vector of parameters **β*_1_*** and dummy-coded variable ***Textbook dummies_*j*_***, describing the average difference in the intercept compared to the average intercept of the reference group if school *j* uses a textbook different from the reference textbook; ***W****_*j*_***β**_2_, where **β**_2_ is a vector of parameters describing the average difference in the intercept for the standardized values of the covariates in ***W****_*j*_* measured for school *j*. ***W****_*ij*_***β***_3_*, where **β**_3_ is a vector of parameters describing the average difference in the intercept for the standardized values of the covariates in ***W*_*ij*_** measured for student *i* in school *j*; *u_*j*,__0_*, the residual for school *j* when *t* = 0; and the error term *e_*ij*,__0_* for person *i* in school *j* when *t* = 0.

*SL*_*ij*_ is a function of: parameter λ_0_, the average of the change scores across all schools and students; a vector of parameters **λ***_1_* and dummy-coded variable *Textbook dummies_*j*_*, describing the average difference in the change score compared to the average change score of the reference group if school *j* uses a textbook different from the reference textbook; ***W****_*j*_***λ**_2_, where λ_2_ is a vector of parameters describing the average difference in the change score for the standardized values of the covariates in ***W****_*j*_* measured for school *j*.***W****_*ij*_***λ***_3_*, where **λ**_3_ is a vector of parameters describing the average difference in the change score for the standardized values of the covariates in ***W****_*ij*_* measured for student *i* in school *j*; *u_*j*,__1_*, the change score residual for school *j* when *t* = 1; and the change score error term *e_*ij*,__1_* for person *i* in school *j* when *t* = 1. We assume that the level 1 errors, *e_*ij*,__0_* and *e_*ij*,__1_*, have a multivariate normal distribution:

(5) $$\left(\begin{array}{c} e_{i\,j,O}\\ e_{i\,j,1} \end{array}\right)∼N\,\left(\left(\begin{array}{c} 0\\ 0 \end{array}\right),\left(\begin{array}{cc} \sigma_{e_{i\,j,O}}^{2}\; & \sigma_{\sigma_{e_{O}\,e_{1}}}\\ \sigma_{e_{O}\,e_{1}}\; & \sigma_{e_{i\,j,1}}^{2} \end{array}\right)\right)$$

Furthermore, we assume that the level 2 errors, *u_*j*,__0_* and *u_*j*,__1_*, have a multivariate normal distribution:

(6) $$\left(\begin{array}{c} u_{j,O}\\ u_{j,1} \end{array}\right)∼N\,\left(\left(\begin{array}{c} 0\\ 0 \end{array}\right),\left(\begin{array}{cc} \sigma_{u_{j,O}}^{2}\; & \sigma_{\sigma_{u_{O}\,u_{1}}}\\ \sigma_{u_{O}\,u_{1}}\; & \sigma_{u_{j,1}}^{2} \end{array}\right)\right)$$

However, change score models for describing change between two time points have a well-known limitation (McArdle, 2009; Castro-Schilo and Grimm, 2018). This limitation is different from the autoregression model, but it is also caused by the measurement error and sampling error in the two academic performance measurements. In this case, because the change score is inferred from the academic performance measurements, the change score “inherits” the measurement error and sampling error of both measurements. Accordingly, the change score is unreliable. Therefore, even if an effect of the independent variable on learning progress exists, this effect may not be significant on the change score.

The choice between the autoregression model and the change score model can therefore be characterized as a choice between “a biased but efficient estimate” versus “an unbiased but inefficient estimate.” Accordingly, a autoregression model may erroneously lead to finding a tangible effect, whereas the change score model may erroneously lead to rejecting a tangible effect. This is often referred to as “Lord’s Paradox” (e.g., Allison, 1990; Castro-Schilo and Grimm, 2018, pp. 37–38; Eriksson and Häggström, 2014; Gollwitzer et al., 2014, pp. 676–678; McArdle, 2009; van Breukelen, 2013, pp. 898–900). Hence, often the autoregression model finds a significant effect, whereas the change score model does not. In this case it is unknown whether this is due to the autoregression model estimates being biased, or the change score model being too conservative. However, if both models “agree” in their results, it is more plausible that the results are not an artifact due to biased estimates or a lack of statistical power. Therefore, we used both models, as a way of testing the robustness of our results.

### Missing Data

In our sample 2.06% of the data was missing on average (see Table 2). Hence, we used full information maximum likelihood to attain unbiased and efficient estimates for missing values (Schafer and Graham, 2002). With this technique, the handling of the missing data is incorporated into the estimation technique of full information maximum likelihood (FIML, Enders and Bandalos, 2001; Raykov, 2005). This method will yield unbiased estimates of the parameters and standard errors if the missing values are missing at random on the variables that are part of the analysis. Therefore, the variances of the independent variables were always freely estimated, and they were allowed to freely correlate. This does not apply to the four textbook dummy variables which had no missing values.

**TABLE 2:** Descriptive statistics across textbooks.

**Book**	***N*_Schools_**	***N*_Students_**	**Perf.**		**Perf.**		**Eng.**		**Eng.**		**SES^3^**		**Other**	
			**Grade 6**		**Grade 4**		**Grade 6^1^**		**Grade 4^2^**				**language^4^**	
								
			***M***	***SD***	***M***	***SD***	***M***	***SD***	***M***	***SD***	***M***	***SD***	***M***	***SD***
1	10	316	593.0	53.3	542.7	53.0	8.7	1.7	9.3	1.8	0.1	1.3	0.2	0.8
2	9	237	579.5	52.8	515.9	56.1	8.3	1.8	9.0	1.9	–0.1	1.4	0.6	1.1
3	10	379	585.5	56.3	530.2	56.0	8.2	1.8	9.0	1.7	0.0	1.2	0.2	1.0
4	60	1888	585.9	54.2	529.9	58.6	8.4	1.8	9.3	1.8	–0.0	1.5	0.3	0.7
5	9	231	580.5	60.0	520.2	64.0	8.6	1.7	9.1	2.0	0.1	1.5	0.4	0.5
Total	98	3051	585.7	54.8	529.4	58.3	8.4	1.8	9.2	1.8	0.0	1.4	0.3	0.8

*1, 62 students (2.03%) had missing values for engagement in sixth grade. Reported statistics for engagement in sixth grade are without these students; 2, 39 students (1.28%) had missing values for engagement in fourth grade. Reported statistics for engagement in fourth grade are without these students; 3, 259 students (8.49%) had missing values for SES. Reported statistics for SES are without these students; 4, 17 students (0.56%) had missing values for other language. Reported statistics for SES are without these students.*

## Results

### Descriptive Statistics

Table 2 shows descriptive statistics across textbooks for both measures of academic performance in reading comprehension, both measures of engagement and the control variables. Overall, we see that Textbook 4 is used in the majority of schools (61.22%), the other four textbooks are used in only nine (9.18%) or ten schools (10.20%). Overall, in the fourth grade Textbook 1 and Textbook 2 show the largest difference in academic performance, they have the highest average academic performance and lowest average academic performance, respectively. Accordingly, the group of students with Textbook 1 has the lowest proportion of students who speak another language at home and the highest average SES, whereas the group of students with Textbook 2 has the highest proportion of students who speak another language and the lowest average SES. In the sixth grade the difference in reading comprehension between these two groups is smaller.

Table 3 shows the matrices of the zero-order correlation coefficients at the student-level and school-level between academic performance in the fourth grade, academic performance in the sixth grade, engagement in the fourth grade, engagement in the sixth grade, socioeconomic status and other language. At both levels, academic performance in sixth grade is significantly related to academic performance in fourth grade, socioeconomic status and other language. Engagement in sixth grade is significantly related to engagement in fourth grade at both the school-level and student-level, and it is also significantly related to academic performance in the fourth grade and sixth grade at the student level.

**TABLE 3:** Matrix of the zero-order correlation coefficients at the student-level and school-level.

**Variables**	**1.**	**2.**	**3.**	**4.**	**5.**	**6.**	***M***	***SD***
**Student level**								
1. Performance grade 6	–						NA	51.95
2. Performance grade 4	0.56^∗^	–					NA	54.57
3. Engagement grade 6	0.06^∗^	0.05^∗^	–				NA	1.55
4. Engagement grade 4	0.07^∗^	0.04	0.27^∗^	–			NA	1.11
5. Socioeconomic status	0.30^∗^	0.31^∗^	0.03	–0.02	–		NA	1.33
6. Other language	−0.10^∗^	−0.15^∗^	0.01	0.01	−0.17^∗^	–	NA	0.37
**School level**								
1. Performance grade 6	–						585.11	19.92
2. Performance grade 4	0.78^∗^	–					528.12	21.21
3. Engagement grade 6	–0.12	–0.03	–				9.53	0.39
4. Engagement grade 4	0.15	0.14	0.50^∗^	–			8.90	0.43
5. Socioeconomic status	0.72^∗^	0.67^∗^	–0.14	–0.11	–		–0.03	0.53
6. Other language	−0.46^∗^	−0.68^∗^	0.00	−0.27^∗^	–0.24	–	0.24	0.21

*^∗^Zero order correlation is significant at the 0.05 level (2-tailed). NA = not applicable to the student-level which is centered on the cluster-mean.*

### Academic Performance in Reading Comprehension

#### Results Multilevel Autoregression Model

Table 4 shows the four multilevel autoregression models that were fit to assess the effects of textbooks on academic performance in reading comprehension. The results of the null model show that the majority of the variance in academic performance for reading comprehension is at the student-level. In Models 1, 2 and 3 the average effects of the dummy textbook variables, compared to the average performance of the students who have the reference textbook, are shown.

**TABLE 4:** Coefficients multilevel autoregression model for reading comprehension.

**Fixed effects**	**Null model**		**Model 1**		**Model 2**		**Model 3**	
	**Effect**	**SE**	**Effect**	**SE**	**Effect**	**SE**	**Effect**	**SE**
Intercept	585.0	2.4	585.1	3.2	585.1	2.3	585.6	2.2
**Student level predictors**								
Performance fourth grade					0.5	0.0	0.5	0.0
Engagement							1.1	0.6
Socioeconomic status							5.6	0.7
Other language							–0.4	2.0
**School level predictors**								
Average performance fourth grade					0.8	0.1	0.4	0.4
Average engagement							–2.5	7.8
Average socioeconomic status							16.1	8.3
Percentage other language							–16.6	26.6
Textbook 1: *Kameleon*			–8.9	6.6	–1.5	5.5	–1.1	5.0
Textbook 2: *Taalbende*			–7.7	7.4	6.1	6.0	5.1	5.0
Textbook 3: *Taalsignaal*			0.3	5.8	0.5	4.7	–0.8	4.7
Textbook 4: *Tijd voor taal (ref.)*								
Textbook 5: *Totemtaal*			–3.5	8.4	3.4	5.1	–0.1	5.1
**Random effects variance**								
Student level	2698.0	97.5	2699.1	97.0	1844.5	68.7	1792.3	66.2
School level	397.1	76.2	383.1	75.4	155.6	41.6	124.0	42.6
**Model fit**								
Log-likelihood	−16461.045		−16459.582		−32468.603		−44045.461	
χ^2^	0.009		0.007		0.000		46.799	
df	0		0		0		16	
CFI	1		1		1		0.981	
RMSEA	0		0		0		0.025	
	ICC		*R*^2^		*R*^2^		*R*^2^	
Student level	87.17%		−0.04%		31.63%		33.57%	
School level	12.83%		3.54%		60.82%		68.77%	

*ref, reference.*

In Model 1, none of the textbook dummy variables has a significant effect on the average academic performance. Together, the textbook dummy variables only explain 3.54% of the variance between schools. The pairwise contrasts between the estimated effects of textbook dummy variables (not shown in Table 4) do not show any significant difference either.

In Model 2, academic performance in the fourth grade significantly and positively predicts academic performance in the sixth grade at both the student-level and school-level. Again, none of the textbook dummy variables have a significant effect on the average academic performance. The pairwise contrasts between the estimated effects of textbook dummy variables do not show any significant difference. The variables in this model explain 31.63% of the variance between students and 60.82% of the variance between schools.

In Model 3, academic performance in the fourth grade and socio-economic status significantly positively predict academic performance in the sixth grade. Engagement in the fourth grade and other language have no significant effect at both the student-level and school-level. Again, none of the textbook dummy variables has a significant effect on the average academic performance. The pairwise contrasts between the estimated effects of textbook dummy variables do not show any significant difference. The variables in this model explain 33.57% of the variance between students and 68.77% of the variance between schools.

#### Results Multilevel Change Score Model

Table 5 shows the four multilevel change score models that were fit to assess the effects of textbooks on learning progress in reading comprehension. The results of the null model show that the majority of the variance in the intercept (86.95%) and slope (92.88%) are at the student-level. There is also a negative correlation between the random effects of the intercept and slope at the student-level (−0.51) and school-level (−0.41). This means that students/schools with lower reading comprehension in Grade 4 tend to have a steeper growth between fourth and sixth grade. In Models 1, 2, and 3, the average effects of textbook dummy variables on the intercept and slope, compared to the average intercept and slope of students who have the reference textbook, are shown.

**TABLE 5:** Coefficients multilevel change score model for reading comprehension.

**Fixed effects**	**Null model**		**Model 1**		**Model 2**		**Model 3**	
	**Effect**	**SE**	**Effect**	**SE**	**Effect**	**SE**	**Effect**	**SE**
Intercept mean	528.1	2.5	529.1	3.1	529.4	2.3	529.4	2.3
Slope mean	56.9	2.3	56.0	2.8	56.6	2.8	57.2	2.5
**Student level predictors intercept**								
Engagement					1.5	0.8	1.5	0.8
Socioeconomic status					12.0	0.9	12.0	0.9
Other language					–14.7	2.9	–14.7	2.9
**Student level predictors slope**								
Intercept							–0.5	0.0
Engagement					0.4	0.8	1.1	0.6
Socioeconomic status					–0.5	0.9	5.6	0.7
Other language					7.0	2.4	–0.4	2.0
**School level predictors intercept**								
Average engagement					0.3	6.2	0.4	6.3
Average socioeconomic status					21.1	4.2	21.0	4.3
Percentage other language					–55.8	9.9	–54.4	9.8
Textbook 1: *Kameleon*			14.1	6.4	5.2	4.8	5.2	4.8
Textbook 2: *Taalbende*			–18.2	7.3	–2.1	6.1	–2.0	6.0
Textbook 3: *Taalsignaal*			–0.3	5.4	–4.0	4.2	–4.2	4.2
Textbook 4: *Tijd voor taal*								
Textbook 5: *Totemtaal*			–9.0	11.9	–0.1	7.5	–0.2	7.6
**School level predictors slope**								
Intercept							–0.5	0.2
Average engagement					–2.7	7.8	–2.6	7.9
Average socioeconomic status					2.8	3.6	13.7	6.2
Percentage other language					24.3	10.0	–3.9	15.5
Textbook 1: *Kameleon*			–5.0	6.1	–3.6	6.0	–0.9	4.9
Textbook 2: *Taalbende*			10.4	5.7	6.0	5.4	5.0	5.1
Textbook 3: *Taalsignaal*			0.6	5.0	1.3	5.0	–0.9	4.8
Textbook 4: *Tijd voor taal*								
Textbook 5: *Totemtaal*			5.5	6.7	0.1	6.7	–0.1	5.1
**Random effects student level**								
Intercept	2977.3	84.1	2976.6	84.0	2658.2	78.7	2658.2	78.7
Slope	2488.5	92.5	2487.7	92.4	2479.3	93.6	1792.4	66.2
Intercept × slope	–1384.0	76.0	–1383.2	76.0	–1350.8	73.4	*NA*	*NA*
**Random effects school level**								
Intercept	446.9	91.5	393.1	79.3	115.2	34.4	115.1	34.4
Slope	190.8	48.5	178.4	45.1	155.0	43.4	123.9	42.6
Intercept × slope	–119.9	49.7	–94.9	46.5	–59.4	26.8	NA	NA
**Model fit**								
Log-likelihood	−32474.524		−32468.603		−44066.269		−44066.269	
χ^2^	0.001		0.002		43.215		43.224	
df	0		0		12		12	
CFI	1.000		1.000		0.981		0.981	
RMSEA	0.000		0.000		0.029		0.029	

In Model 1, two textbook dummy variables have a significant effect on the intercept, whereas no textbook dummy variable has a significant effect on the slope. Schools using textbook 1, tend to have higher reading comprehension scores in Grade 4 than schools using the reference textbook. Schools using textbook 2 tend to have lower reading comprehension scores in Grade 4. Together, the textbook dummy variables only explain 12.04% of the variance in the intercepts at the school-level and 6.50% of the variance in the slopes at the school-level. The pairwise contrasts between the estimated effects of textbook dummy variables on the slope (not shown in Table 5) do not show any significant difference.

In Model 2, socioeconomic status and other language significantly predict the intercept at both the student-level and school-level. However, only at the school-level do socioeconomic status and other language significantly predict the slope, not at the student level. None of the textbook dummy variables have a significant effect on the intercept or slope. The pairwise contrasts between the estimated effects of textbook dummy variables on the slope do not show any significant difference either. Together, the variables explain 10.66% of the variance in the intercepts and 0.37% of the variance in the slopes at the student-level. At the school-level, the variables explain 73.69% of the variance in the intercepts and 19.18% of the variance in the slopes.

In Model 3, the intercept significantly predicts the slope at both the student-level and school-level. Socioeconomic status and other language significantly predict the intercept at the student-level and school-level. Only socioeconomic status significantly predicts the slope at the student-level and school-level, other language does not. None of the textbook dummy variables have a significant effect on the intercept or slope. The pairwise contrasts between the estimated effects of textbook dummy variables on the slope do not show any significant difference either. Together, the variables explain 10.66% of the variance in the intercepts and 28.01% of the variance in the slopes at the student-level. At the school-level, the variables explain 73.69% of the variance in the intercepts and 35.22% of the variance in the slopes.

### Engagement in Reading Comprehension

#### Results: Multilevel Autoregression Model

Table 6 shows the four multilevel autoregression models that were fit to assess the effects of textbooks on students’ reading engagement. The results of the null model show that the majority of the variance in engagement is at the student-level. In Models 1, 2, and 3 the average effects of the dummy textbook variables, compared to the average engagement of the students who have the reference textbook, are shown.

**TABLE 6:** Coefficients multilevel autoregression model for reading engagement.

**Fixed effects**	**Null model**		**Model 1**		**Model 2**		**Model 3**	
	**Effect**	**SE**	**Effect**	**SE**	**Effect**	**SE**	**Effect**	**SE**
Intercept	8.91	0.05	8.97	0.06	8.91	0.06	8.91	0.06
**Student level predictors**								
Engagement fourth grade					0.19	0.01	0.19	0.01
Performance fourth grade							0.00	0.00
Socioeconomic status							–0.03	0.02
Other language							0.02	0.05
**School level predictors**								
Average engagement fourth grade					0.53	0.19	0.51	0.20
Average performance fourth grade							0.00	0.01
Average socioeconomic status							–0.13	0.14
Percentage other language							–0.46	0.42
Textbook 1: *Kameleon*			0.06	0.14	0.01	0.14	–0.01	0.13
Textbook 2: *Taalbende*			–0.23	0.18	–0.06	0.18	–0.02	0.15
Textbook 3: *Taalsignaal*			–0.17	0.18	0.09	0.15	0.01	0.15
Textbook 4: *Tijd voor taal*								
Textbook 5: *Totemtaal*			–0.36	0.08	–0.13	0.11	–0.06	0.13
**Random effects variance**								
Student level	1.22	0.07	1.22	0.07	1.13	0.06	1.14	0.06
School level	0.18	0.04	0.17	0.03	0.14	0.03	0.12	0.03
**Model fit**								
Log-likelihood	−4560.765		−4557.596		−9489.391		−32672.141	
χ^2^	0.000		0.000		0.001		46.501	
df	0		0		0		16	
CFI	1.000		1.000		1.000		0.954	
RMSEA	0.000		0.000		0.000		0.025	
	ICC		*R*^2^		*R*^2^		*R*^2^	
Student level	87.14%		0.00%		7.38%		6.56%	
School level	12.86%		5.56%		22.22%		33.33%	

In Model 1, none of the textbook dummy variables has a significant effect on the average engagement. There are two pairwise contrasts between the estimated effects of textbook dummy variables that are significant. There is a significant difference between textbook 1 and textbook 5 (−0.417, *p* = 0.002), and between textbook 4 and textbook 5 (−0.362, *p* = 0.000). The effect sizes of both significant contrasts are small. Together, the textbook dummy variables only explain 5.56% of the variance between schools.

In Model 2, students’ engagement in the fourth grade significantly and positively predicts students’ engagement in the sixth grade at both the student-level and school-level. None of the textbook dummy variables have a significant effect on the average engagement. The variables in this model explain 7.38% of the variance between students and 22.22% of the variance between schools.

In Model 3, only engagement in the fourth grade significantly and positively predicts engagement in the sixth grade at both the student-level and school-level. Performance in the fourth grade, SES and other language have no significant effect at both the student-level and school-level. Again, none of the textbook dummy variables has a significant effect on engagement. The pairwise contrasts between the estimated effects of textbook dummy variables do not show any significant difference. The variables in this model explain 6.56% of the variance between students and 33.33% of the variance between schools.

#### Results: Multilevel Change Score Model

Table 7 shows the four multilevel change score models that were fit to assess the effects of textbooks on the evolution in engagement. The results of the null model show that the majority of the variance in the intercept (94.09%) and slope (94.08%) is at the student-level. There is also a negative correlation between the random effects of the intercept and slope at the student-level (−0.76) and school-level (−0.44). In Models 1, 2, and 3, the average effects of textbook dummy variables on the intercept and slope, compared to the average intercept and slope of students who have the reference textbook, are shown.

**TABLE 7:** Coefficients multilevel change score model for reading engagement.

**Fixed effects**	**Null model**		**Model 1**		**Model 2**		**Model 3**	
	**Effect**	**SE**	**Effect**	**SE**	**Effect**	**SE**	**Effect**	**SE**
Intercept mean	9.53	0.05	9.63	0.06	9.63	0.06	9.63	0.06
Slope mean	–0.62	0.05	–0.66	0.07	–0.67	0.07	–0.63	0.06
**Student level predictors intercept**								
Reading comprehension 2016					0.00	0.00	0.00	0.00
Socioeconomic status					0.02	0.03	0.02	0.03
Other language					0.08	0.10	0.08	0.10
**Student level predictors slope**								
Intercept							–0.81	0.01
Reading comprehension 2016					0.00	0.00	0.00	0.00
Socioeconomic status					–0.05	0.03	–0.03	0.02
Other language					–0.05	0.10	0.02	0.05
**School level predictors intercept**								
Reading comprehension 2016					0.00	0.01	0.00	0.01
Average socioeconomic status					–0.13	0.16	–0.13	0.16
Percentage other language					0.24	0.45	0.24	0.45
Textbook 1: *Kameleon*			0.11	0.18	0.14	0.19	0.14	0.19
Textbook 2: *Taalbende*			–0.32	0.14	–0.37	0.15	–0.37	0.15
Textbook 4: *Taalsignaal*			–0.44	0.13	–0.42	0.13	–0.42	0.13
Textbook 6: *Tijd voor taal*								
Textbook 8: *Totemtaal*			–0.44	0.16	–0.47	0.17	–0.47	0.17
**School level predictors slope**								
Intercept							–0.45	0.22
Reading comprehension 2016							0.00	0.01
Average socioeconomic status							–0.13	0.14
Percentage other language							–0.48	0.43
Textbook 1: *Kameleon*			–0.06	0.20	–0.10	0.19	–0.03	0.14
Textbook 2: *Taalbende*			0.09	0.18	0.20	0.17	0.04	0.16
Textbook 4: *Taalsignaal*								
Textbook 6: *Tijd voor taal*			0.29	0.12	0.26	0.12	0.07	0.15
Textbook 8: *Totemtaal*			0.07	0.15	0.23	0.19	0.02	0.17
**Random effects student level**								
Intercept	2.39	0.09	2.39	0.09	2.38	0.09	2.38	0.09
Slope	2.70	0.10	2.70	0.10	2.70	0.10	1.14	0.06
Intercept × slope	–1.94	0.07	–1.93	0.07	–1.93	0.07		
**Random effects school level**								
Intercept	0.15	0.03	0.12	0.03	0.11	0.03	0.11	0.03
Slope	0.17	0.04	0.16	0.04	0.14	0.04	0.12	0.03
Intercept × slope	–0.07	0.03	–0.05	0.03	–0.05	0.03		
**Model fit**								
Log-likelihood	−10106.581		−10097.279		−32672.141		−32672.141	
χ^2^	0.000		0.000		27.851		27.850	
df	0		0		12		12	
CFI	1.000		1.000		0.976		0.976	
RMSEA	0.000		0.000		0.021		0.021	

In Model 1, three textbook dummy variables have a significant effect on the intercept, whereas only one textbook dummy variable has a significant effect on the slope. This effect on the slope has a non-meaningful effect size. Together, the textbook dummy variables only explain 0.04% of the variance in the intercepts at the school-level and 0.04% of the variance in the slopes at the school-level. The pairwise contrasts between the estimated effects of textbook dummy variables on the slope (not shown in Table 7) do not show any significant difference.

In Model 2, none of the control variables significantly predict the intercept and slope at the student-level and school-level. Three textbook dummy variables have a significant effect on the intercept, whereas only one textbook dummy variable has a significant effect on the slope. The pairwise contrasts between the estimated effects of textbook dummy variables on the slope do not show any significant difference. Together, the variables explain 0.38% of the variance in the intercepts and 0.22% of the variance in the slopes at the student-level. At the school-level, the variables explain 27.81% of the variance in the intercepts and 15.48% of the variance in the slopes.

In Model 3, the intercept significantly predicts the slope at both the student-level and school-level. None of the control variables significantly predict the intercept and slope at the student-level and school-level. Three textbook dummy variables have a significant effect on the intercept, whereas none of the textbook dummy variables have a significant effect on the intercept or slope. The pairwise contrasts between the estimated effects of textbook dummy variables on the slope do not show any significant difference either. Together, the variables explain 0.38% of the variance in the intercepts and 58.03% of the variance in the slopes at the student-level. At the school-level, the variables explain 27.81% of the variance in the intercepts and 28.57% of the variance in the slopes.

## Discussion

In this study, we investigated whether a school’s choice for a textbook affects children’s learning progress in reading comprehension and evolution in engagement in reading from the fourth until the sixth grade in primary education. We compared five textbooks by estimating contrasts based on a multilevel autoregression model and a multilevel change score model. Both models were estimated with and without controls for student background characteristics.

We do not find support for our hypotheses, for none of the estimated contrast in the final models were significant. Accordingly, we cannot reject the null hypotheses that the textbooks do not affect children’s learning progress in reading comprehension and engagement in reading. These results are somewhat surprising, given that former research on the effects of textbooks generally found significant effects for academic performance (Goffin et al., 2016; Bellens et al., 2019 (e.g., Törnroos, 2005; Van Steenbrugge et al., 2013; Hadar, 2017; van den Ham and Heinze, 2018). Furthermore, there are also several theoretical reasons to expect that textbooks matter for student’s academic performance (Schmidt et al., 1997; Valverde et al., 2002; Törnroos, 2005) in reading comprehension and engagement. However, it should be noted that we assessed the effects on performance in reading comprehension, whereas former research mainly focused on mathematics. Former research also did not assess the effects of textbooks on engagement. Furthermore, our study mainly assesses the effects on learning progress between the fourth and sixth grade, whereas most former studies only had cross-sectional data. Hence, differences in the outcome and the research strategy may explain why this study did not yield a significant effect of textbooks.

A main reason to conduct this study was the large decrease in average ability in reading comprehension between 2006 and 2016 in Flanders. Because Flemish education is characterized by large educational freedom for schools, the teachers’ daily practices are often based on textbooks. We hypothesized that new textbooks may be lower in quality, which would negatively affect students’ average performance in reading comprehension. However, considering that we do not find differences in learning progress between students who have different textbooks, it is implausible that the new textbooks are responsible for the decrease in average ability.

## Limitations and Suggestions for Future Research

While this study had a relatively large dataset compared to former studies on textbooks, there were still some limitations. When assessing the estimated standard error, it seems that we had just enough data to determine if an effect of a small size (when using Cohen’s d) was significant. However, for any effect smaller than that, we would not have been able to assess its significance. The reason for this limited statistical power was that only in relatively few schools another textbook was used, compared to the textbook that was used in most schools (textbook 4).

Multilevel autoregression models and multilevel change score models are correlational models, which do not allow us to make strong causal claims concerning the effects of textbooks on learning progress. It is likely that some sources of confounding were not controlled for, especially concerning the characteristics of schools and teachers. Hence, if schools and teachers that selected different textbooks also differ on other characteristics that influence learning progress in reading comprehension, our estimated effects will be biased. However, we were limited in how many confounding variables could be controlled for due to the sample size. We do note that, in our dataset, there were no other variables that would tangibly explain academic performance in reading comprehension next to the variables we selected. However, we may have simply lacked the relevant variables to control for.

Our data does seem to improve on the majority of prior studies on the effects of textbooks, for we were able to estimate learning progress, whereas prior studies only had cross-sectional data. However, having more than two repeated measures would also allow for the estimation of growth curves, which would improve the description of learning progress (e.g., Singer and Willett, 2003). This would also reduce the power for estimating the effects of textbooks on learning progress. Relatedly, our study was limited to the learning progress during the last 2 years of primary education. It is not impossible that textbooks matter more during early primary education.

We also note that the inferences in this study cannot be simply generalized to other education systems. Across education systems textbooks are unique, they will differ in their potential influence due to differences in educational freedom and how teachers use them. Therefore, in other education systems different effects of textbooks could be found. Moreover, the findings in this study are only about reading comprehension and we do not know if comparable results would be attained for other outcomes.

## Conclusion

In sum, we do not find that the textbooks affect students’ learning progress in reading comprehension and evolution in engagement for reading during the last 2 years of primary education. Therefore, we cannot reject the null hypothesis that textbooks do not affect learning progress and engagement. Accordingly, Flanders’ decline in students’ average ability in reading comprehension cannot be attributed to specific textbooks that negatively affect learning progress.

## Data Availability Statement

The datasets generated for this study are available on request to the corresponding author.

## Ethics Statement

The studies involving human participants were reviewed and approved by Ethische Commissie (KU Leuven). Written informed consent to participate in this study was provided by the participants’ legal guardian/next of kin.

## Author Contributions

JD performed the analyses, wrote the first draft of the manuscript, and finished the final manuscript. KB and BD gave feedback on the first and second draft of the manuscript and necessary suggestions were made for the literature, Materials and Methods, and Conclusion section.

## Conflict of Interest

The authors declare that the research was conducted in the absence of any commercial or financial relationships that could be construed as a potential conflict of interest.
